# Current Clinical Implications of Frailty and Sarcopenia in Vascular Surgery: A Comprehensive Review of the Literature and Consideration of Perioperative Management

**DOI:** 10.3400/avd.ra.22-00035

**Published:** 2022-09-25

**Authors:** Hiroshi Furukawa

**Affiliations:** 1 Department of Cardiovascular Surgery, Tokyo Women’s Medical University Adachi Medical Center, Tokyo, Japan

**Keywords:** frailty, sarcopenia, vascular surgery, aortic aneurysm, peripheral arterial disease

## Abstract

Frailty is a well-known geriatric syndrome of impaired physiological reserve and increased vulnerability to stressors. Sarcopenia is also used as a parameter of physical impairment characterized by muscle weakness. As population aging has become more prominent in recent years, both modalities are now regarded as clinically important prognostic tools defined by multidimensional factors that may affect clinical outcomes in various clinical settings. A preoperative surgical risk analysis is mandatory to predict clinical and surgical outcomes in all surgical practices, particularly in high-risk surgical patients. In vascular surgical settings, frailty and sarcopenia have been accepted as useful prognostic tools to evaluate patient characteristics before surgery, as these may predict perioperative clinical and surgical outcomes. Although minimally invasive surgical approaches, such as endovascular therapy, and hybrid approaches have been universally developed, achieving good vascular surgical outcomes for high-risk cohorts remains to be challenge due to the increasing prevalence of elderly patients and multiple preoperative co-morbidities in addition to frailty and sarcopenia. Therefore, to further improve clinical and surgical outcomes, these preoperative geriatric prognostic factors will be of great importance and interest in vascular surgical settings for both physicians and surgeons.

## Introduction

Frailty was first described as a “frail elderly” by Rubenstein in 1981^1)^; subsequently, it was defined as a syndrome of impaired physiological reserve and increased vulnerability to stressors. Rockwood et al. introduced a comprehensive geriatric assessment for frailty in elderly cohorts to predict clinical outcomes.^2)^ As Fried et al. proposed the evaluation of frailty using a multifactorial assessment with elements, namely, unintentional weight loss, self-reported exhaustion, a weak grip strength, slow walking speed, and low physical activity,^3)^ the concept of frailty has been attracting increasing clinical attention. When exposed to stressors, patients with frailty are prone to adverse events, procedural complications, prolonged recovery, functional decline, and even mortality.^4)^ Although a consensus has not yet been reached on the best approach to assess frailty in clinical practice, it has been increasingly recognized as an important negative prognostic indicator of poor outcomes in patients undergoing invasive surgery. Therefore, a pre-procedural frailty assessment may facilitate the identification of potentially modifiable factors that may improve the outcomes of patients with frailty.^5)^

Sarcopenia is a newly defined condition that is mainly characterized by muscle volume loss.^6)^ It has been implicated in aging and co-morbidities; it is a well-known key element of frailty and predicts morbidity and mortality in clinical settings.^7)^ The European Working Group on Sarcopenia in Older People established the following criteria to diagnose sarcopenia: a low muscle mass, low muscle strength, and low physical performance.^8)^

**Figure 1:**
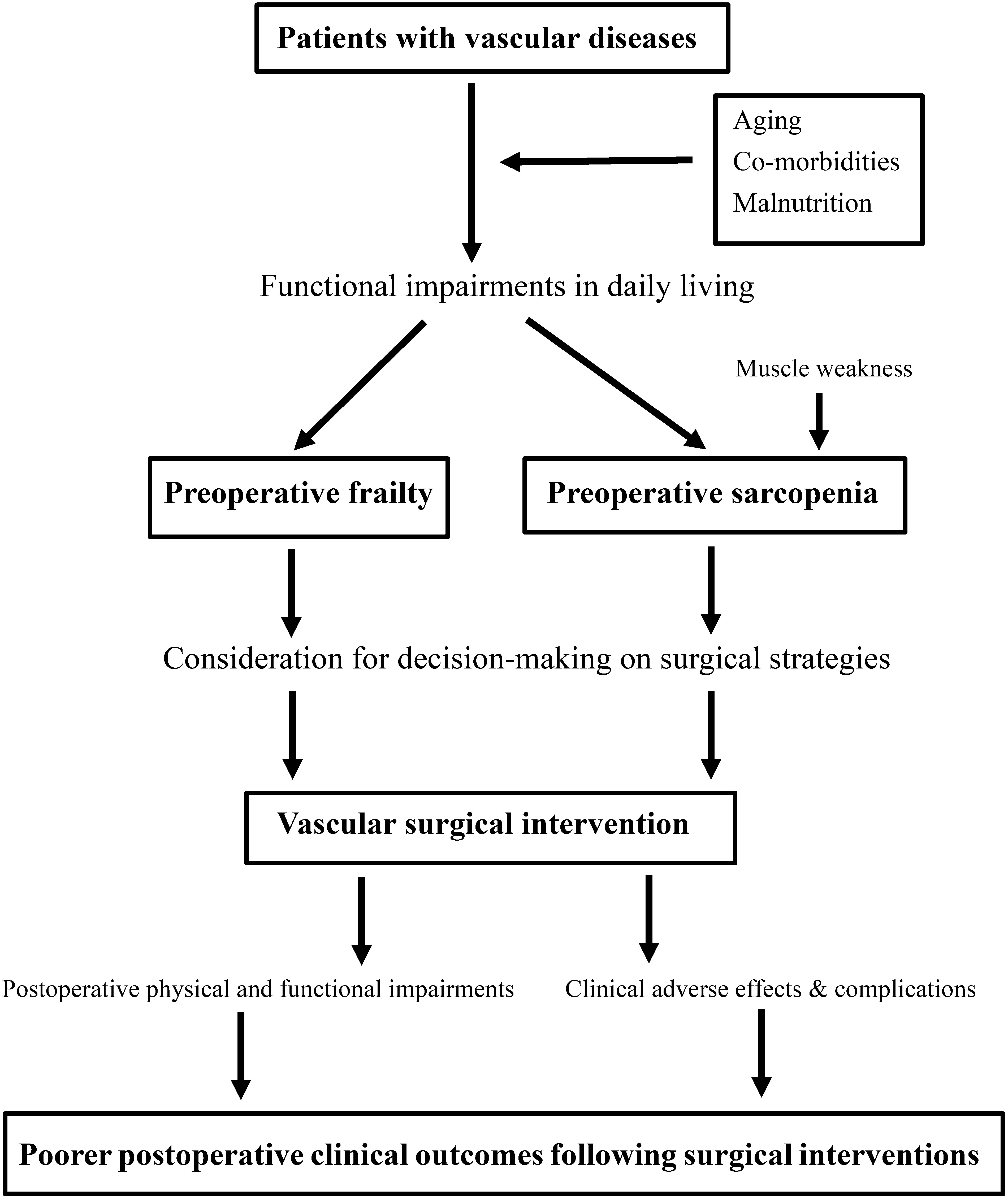
Fig. 1 Clinical algorithm for synergistic adverse effects on preoperative frailty and sarcopenia in vascular surgical settings.

Frailty and sarcopenia may exert synergistic adverse effects that may result in physical and functional impairments in daily life. In the last few decades, research has focused on their ability to predict the clinical and surgical outcomes of cohorts with various vascular diseases.^9–13)^ Furthermore, assessments of frailty and sarcopenia may be an important part of the preoperative decision-making process of surgical strategies in vascular surgery (**Fig. 1**).^10)^

Preoperative frailty and sarcopenia in patients who underwent vascular surgical interventions synergically aggravate postoperative physical functional impairments with adverse effects and complications, which leads to poor postoperative clinical outcomes.

To confirm the accurate assessment of frailty or sarcopenia in vascular surgical settings, we herein summarized and highlighted their clinical impact. Due to the nature of this review, there are no ethical concerns and neither informed consent nor Institutional Review Board approval required.

## General Concept and Definition of Frailty and Sarcopenia in Surgical Settings

The original clinical evaluation of frailty using an eyeball assessment by medical staff was subjective and hard to define objectively; therefore, precise objective clinical tools have since been developed to accurately define frailty. Representative and available scales with the potential to detect and evaluate frailty or sarcopenia are shown in **Table 1**.

**Table 1:** Table 1 Clinical indicators of frailty and sarcopenia in vascular surgery

Category	Subcategory	Indicator	Criteria, contents, and concept	References
Frailty	Aging	Age	Elderly cohorts (>70 or 75 years old, depending on each study)	32), 35), 37)
	Sex	Female	N/A	37), 60)
	Comorbidity	Cerebrovascular disease	History of major stroke	12), 32), 35), 37)
		Chronic kidney disease	>Class IIIa or >Class IIIb or dialysis dependency	35), 37)
		Cognitive impairment: MoCA	30-point assessment to detect cognitive impairment	24)
		RAI	Frailty screening tool based on the accumulated deficit model	31)
	ADL	Katz score (index)	Independence in feeding, bathing, dressing, transferring, toileting, and urinary continence	36)
		Barthel Index	10-item scale that assesses a patient’s ability to feed, groom, use the toilet, dress, walk, transfer, and climb stairs, as well as fecal incontinence and urinary incontinence	61), 62)
		Functional status	Dependent or independent in daily living, ambulatory or non-ambulatory	12), 25), 37), 50), 55), 57), 58), 64), 65)
		Functional impairment score	Residents’ need for assistance with self-care activities, walking, and locomotion	59)
	Comprehensive	Modified Frailty Index	11-point from the CSFA frailty index	23), 26), 45), 47), 60), 63), 67), 68)
		CFS	CFS classifies frailty using a 9-point scale, with frail patients being defined as those assigned a CFS score ≥5	28), 66)
		GFI	16 items in the domains of functioning	9), 30)
		AVFS	Six variables: emergency admission, dependent mobility, polypharmacy, history of multiple falls, anemia on admission, nutrition score	11), 46)
	Physical status	Weight loss	N/A	27)
		BMI	<18.5 kg/m^2^	32), 35), 37), 61)
		Slow gait speed	N/A	27)
	Nutrition	Anemia	Institutional criteria	32), 35)
		Hypoalbuminemia	Institutional criteria	32), 35), 61), 78)
		GNRI	GNRI=14.89×albumin (g/dL)+41.7×(body weight/ideal body weight)	65), 79)
		Nutritional impairment	Malnutrition (PG-SGASF ≥4 points)	12), 77)
	Muscle strength	Hand grip strength	N/A	27)
Sarcopenia	Muscle volume	PMA, SMA, TPA (TPAI), TAMA, TSMA	Standardized psoas area or total psoas muscle area at the L3 or L4 level by CT measurements	22), 32), 33), 34), 35), 36), 44), 48), 49), 51), 52), 53), 54), 56), 65), 69), 70), 72)
		PLVI	Cross-sectional areas of the bilateral psoas muscles and vertebral body at the L4 level	71)
		Thoracic muscle mass	Measured on axial images at the Th12 level	39)

ADL: activity of daily living; MoCA: Montreal Cognitive Assessment; RAI: Risk Analysis Index; CFS: Clinical Frailty Scale; GFI: Groningen Frailty Index; AVFS: Addenbrookes Vascular Frailty Score; BMI: body mass index; GNRI: Geriatric Nutritional Risk Index; PMA: psoas muscle area; SMA: skeletal muscle area; TPA: total psoas area; TPAI: Total Psoas Area Index; TAMA: total abdominal muscle area; TSMA: total skeletal muscle area; PLVI: Psoas Lumbar Vertebral Index; N/A: not applicable; CSFA: Clinical Frailty Scale algorithm; PG-SGA SF: Patient-Generated Subjective Global Assessment Short Form; CT: computed tomography; Th12: 12th thoracic vertebra level

Frailty was previously identified as a surgical risk factor for transcatheter aortic valve replacement patients^14,15)^; thus, a novel surgical risk analysis tool was developed as an important factor to identify high-risk patients before cardiothoracic^16–19)^ and vascular surgery.^9,10)^ Sarcopenia was initially used as a risk factor in the field of liver transplantation,^20,21)^ and it subsequently became a prognostic factor for a poor outcome after surgical interventions in patients with critical limb ischemia (CLI).^22)^ Therefore, sarcopenia and frailty have become important preoperative prognostic tools in various surgical settings.

## Frailty and Sarcopenia in General Vascular Surgery

The clinical role of frailty and sarcopenia in general and comprehensive vascular surgery has been discussed since 2013. Karam et al. have initially indicated that the simplified Canadian Study of Health and Aging Frailty Index (FI), which is assessed based on easily identifiable patient characteristics, has allowed for the accurate prediction of postoperative morbidity and mortality in patients undergoing vascular surgery.^23)^ Meanwhile, Partridge et al. applied the combination of cognitive impairment or dementia and frailty to patients with abdominal aortic aneurysm (AAA) or peripheral arterial disease (PAD). The combined assessment of frailty and cognition was found to be predictive of adverse postoperative outcomes and a longer hospital stay.^24)^ In 2015, Ambler et al. demonstrated, using the Addenbrookes Vascular Frailty Score, that frailty in vascular surgical patients predicted poorer outcomes.^11)^ Scarborough et al. described the clinical impact of functional dependency, which is observed in patients with frailty and/or sarcopenia. Following major surgery, including vascular surgery, preoperative functional dependency was identified as an independent risk factor for mortality.^25)^ In 2016, Arya et al. introduced the modified FI (mFI) to evaluate preoperative frailty and stated that frail home-dwelling patients undergoing elective vascular procedures were at a high risk of nonhome discharge.^26)^

O’Neill et al. have used the domains nutritional status by weight loss, self-reported exhaustion, slow gait speed, grip strength, and low weekly energy expenditure to assess frailty and found that this clinical evaluation was a useful screening tool to identify frail patients in preoperative assessments.^27)^ In 2018, Donald et al. showed that the Clinical Frailty Scale (CFS) score may be used to predict a discharge to a nursing facility or death after surgical interventions.^28)^ Drudi et al. recently employed multifactorial aspects to analyze the clinical efficacy of frailty, including several prognostic models and scales, in vascular surgery patients.^29)^ By using the Groningen Frailty Index, Visser et al. demonstrated that frailty was associated with a higher risk of postoperative complications and discharge to a nursing home after vascular surgery.^30)^ Rothenberg et al. recently developed a frailty assessment using a risk analysis index, which may be an accurate predictor of mortality for all vascular surgery patients.^31)^

## Frailty and Sarcopenia in Thoracic Aortic Surgery (TAS)

The concept of frailty and sarcopenia has been of great importance in TAS, as surgical interventions for open TAS are some of the most invasive procedures in vascular surgery. Moreover, the development of diagnostic tools, such as computed tomography (CT) and magnetic resonance imaging, has facilitated the detection of thoracic aortic aneurysm (TAA) as well as thoracic aortic dissection.

Ganapathi et al. initially reported the clinical role of frailty in proximal aortic surgery,^32)^ and its importance in TAS has been increasingly recognized. They evaluated the role of frailty using the following comprehensive components (frail score): age >70 years, body mass index (BMI) <18.5 kg/m^2^, anemia, a history of stroke, hypoalbuminemia, and total psoas volume in the bottom quartile of the population. In their study, 25.7% of patients were determined to have preoperative frailty as an independent predictor of the discharge disposition as well as early and late mortality risks. Ikeno et al. recently identified sarcopenia as a predictive tool using the psoas muscle area (PMA) index, defined as PMA at the third lumbar vertebrae (L3) level on CT/body surface area, but not a predictor of hospital death following total arch replacement (TAR) for TAA.^33)^ In 2018, a number of studies were published on frailty and sarcopenia in TAS. Tanaka et al. showed that preoperative sarcopenia defined by the total psoas area (TPA) index correlated with postoperative adverse events, such as organ complications and long-term mortality, after descending TAA repair, including open and endovascular surgeries.^34)^ In a study by Gomibuchi et al., preoperative frailty was defined by age >70 years old, BMI <18.5 kg/m^2^, serum creatinine >1.2 mg/dL, anemia, a history of stroke, and hypoalbuminemia, and the PMA index was used as an independent predictor of the risk of late mortality in patients undergoing acute type A aortic dissection (AAAD) surgery.^35)^ Risk stratifications based on preoperative frailty may affect the mid-term clinical outcomes of patients who underwent elective TAR, as has been indicated by Hiraoka et al.^36)^ Similar findings showed that preoperative frailty defined by six original comprehensive components had potential as a prognostic factor for delays in the recovery of the activities of daily living (ADL) following AAAD surgery, but it did not influence early and mid-term clinical outcomes.^37)^ Surgical interventions for AAAD patients are for life rescue; therefore, a diagnosis of preoperative frailty or sarcopenia is difficult prior to surgery and appears to be meaningless in clinical settings. A recent evaluation of deferral considerations for AAAD surgery revealed that an important factor was preoperative frailty.^38)^ Prognostic factors have the potential to decide the appropriate surgical strategy for AAAD patients, namely, whether to perform minimal invasive ascending aortic replacement for life rescue or extended TAR with or without the frozen elephant trunk technique for a better prognosis.

Panthofer et al. recently reported the derivation and validation of “thoracic sarcopenia” in patients undergoing thoracic endovascular aortic repair (TEVAR) by using muscle mass at the 12th thoracic vertebrae level to detect sarcopenia in TAA patients.^39)^ Brooke et al. evaluated high-risk vascular surgical cohorts and performed comparisons between open surgery for TAA (7850 patients) and TEVAR (1914 patients), wherein they found that early hospital discharge was associated with a significantly lower rate of readmission. They emphasized the need for a safe and cost-effective program for frail patients undergoing vascular surgery.^40)^ These novel preoperative prognostic factors will be important to select minimally invasive approaches, such as endovascular therapy (EVT), or more conservative minimally invasive open surgeries with or without a hybrid approach in order to avoid unexpected postoperative complications in these high-risk and complicated cohorts with TAA.^41,42)^

## Frailty and Sarcopenia in Abdominal Aortic Surgery

In abdominal aortic surgery, advances in endovascular aortic repair (EVAR) were noted to improve early survival and reduce postoperative adverse complications.^43)^ Lee et al. advocated the clinical role of frailty as a discriminator of postoperative class IV complications for open and endovascular AAA repair using the following classifications of frailty: mFI, the Lee Cardiac Revised Index, and the American Society of Anesthesiologists physical status classification in 2011.^44)^

In 2015, Arya et al. reported the significant clinical role of frailty in elective AAA repair, with a higher mFI being associated greater mortality and morbidity in patients undergoing elective EVAR and open AAA repair.^45)^ Srinivasan et al. examined 184 patients with ruptured AAA repair using seven components, called the ruptured aneurysm frailty score, wherein it was found that these components were a good predictor of 1-year mortality.^46)^ In comparisons between open surgery and EVAR for AAA, preoperative frailty and sarcopenia were associated with poor survival and clinical outcomes^47–50)^; however, Indrakusuma et al. demonstrated that sarcopenia defined by a low PMA was not associated with survival in patients with asymptomatic AAA.^51)^

Preoperative frailty and sarcopenia have been proposed as important prognostic factors in AAA patients treated by EVAR. In 2016, Hale et al. initially showed that preoperative sarcopenia detected by CT was an important predictor of long-term mortality in these patients.^52)^ However, discrepancies were noted in the findings of two studies that used preoperative sarcopenia detected by CT measurements of PMA. Thurston et al. showed that preoperative sarcopenia defined by preoperative PMA at the L3 level was associated with poorer survival and longer hospital stay following EVAR.^53)^ On the other hand, Newton et al. found that preoperative sarcopenia did not affect the length of hospital stay but was associated with worse long-term survival.^54)^ Harris et al. identified the preoperative functional status as a predictor of major complications and death after EVAR.^55)^ Lindström et al. have recently conducted a retrospective study, and the findings obtained suggested that the development of sarcopenia was able to predict mortality after EVAR.^56)^ Based on these findings, it can be concluded that preoperative frailty or sarcopenia definitely affects the prognosis of patients with AAA undergoing open repair or EVAR.

## Frailty and Sarcopenia in Peripheral Arterial Revascularization

In patients with PAD, frailty and sarcopenia are well-known factors that may affect the outcomes of these patients because they are complicated by various co-morbidities and low physical activities concomitant with these primary vascular diseases. In 2010, the clinical impact of the functional status prior to surgical interventions was initially discussed by two groups, both of which indicated that a preoperative dependent or non-ambulatory status correlated with poor surgical outcomes.^57,58)^ In 2014, the findings of a study by Vogel et al. were determined to be consistent with clinical outcomes using their own functional impairment score, which indicated that open and endovascular procedures for CLI were associated with similar initial declines in the functional status in frailty cohorts.^59)^ In 2016, a novel evaluation using mFI by Brahmbhatt et al. showed that female sex and frailty were both associated with an increased risk of complications and death following infrainguinal vascular procedures with the highest risk, particularly in frail females.^60)^ In 2017, Kodama et al. reported that the Barthel Index (BI) and BMI were independently associated with all-cause mortality after infrainguinal bypass for CLI.^61)^ Mii et al. also indicated that BI at discharge in patients who underwent infrainguinal bypass for CLI correlated with 3-year clinical outcomes, including overall survival and amputation-free survival, while preoperative BI was not a significant predictor of either outcome.^62)^

Fang et al. suggested the potential of mFI to predict the outcome of patients undergoing major lower extremity amputations and in selecting the most appropriate postoperative planning and care.^63)^ Dinga Madou et al. reported that an objective evaluation of the functional status prior to surgery in PAD patients with CLI is necessary when considering EVT in the elderly with a dependent status.^64)^ Morisaki et al. examined preoperative frailty in CLI patients using two or more of the following categories: a low Geriatric Nutritional Risk Index (GNRI), skeletal muscle mass index, and non-ambulatory status; they then concluded that CLI FI was a risk factor for 2-year amputation-free survival after infrapopliteal revascularization.^65)^ In 2018, Takeji et al. introduced the novel modality of a 9-level CFS to evaluate the clinical outcomes of PAD patients with CLI and demonstrated that frailty was independently associated with 2-year overall survival and amputation-free survival in patients with CLI treated with revascularization.^66)^ Ali et al. assessed the clinical impact of mFI with 11 variables among patients undergoing infrainguinal arterial bypass surgery and showed its potential as a valuable tool for identifying patients at a higher risk of developing postoperative complications after lower extremity revascularization (LER).^67)^ Eslami et al. conducted a preoperative frailty assessment and showed that higher mFI was independently associated with higher postoperative mortality and morbidity.^68)^

Sarcopenia was initially described as a prognostic factor for CLI by Matsubara et al.^22)^; thereafter, the concept of sarcopenia in PAD patients who underwent LER has been gaining great interest. They initially evaluated preoperative sarcopenia using the skeletal muscle area on transverse CT scans at the L3 level, identified sarcopenia as a prognostic factor for CLI patients, and indicated that exercise and nutritional interventions with a focus on attenuating sarcopenia are useful treatment options for CLI patients. In the same year, Swanson et al. showed a novel indicator of central sarcopenia, which is defined by the cross-sectional areas of the psoas muscles and L4 vertebral body at the mid-L4 level. They demonstrated that PAD patients had a lower Psoas Lumbar Vertebral Index (PLVI) than patients with AAA; however, PLVI did not correlate with the severity of symptoms.^69)^ In 2017, Matsubara et al. reported a unique impact of preoperative sarcopenia, namely, that a dual diagnosis of sarcopenia and CLI was associated with higher rates of cardiovascular mortality than in a matched population without a diagnosis of sarcopenia in both open surgery and EVT.^70)^ Nyers et al. found that PLVI did not predict amputation-free survival after open surgery or EVT for PAD.^71)^ In 2018, Juszczak et al. have focused on TPA measured on CT angiograms at the L4 level and suggested its potential to identify patients with a shorter life expectancy after LER; however, low TPA was not associated with an increased rate of postoperative complications or a prolonged hospital stay.^72)^

## Potential Treatment or Strategy for Frailty and Sarcopenia in Vascular Surgery

Novel management with potential clinical applications and further perspectives for frailty or sarcopenia patients has been determined to achieve better clinical outcomes following vascular surgery, as shown in **Table 2**.

**Table 2:** Table 2 Potential management or treatment for frailty and sarcopenia

Category	Subcategory	Potential management or treatment for frailty and sarcopenia	References
Exercise	Cardiac rehabilitation	Perioperative CR may improve postoperative physical mobility, functional capacity, fall prevention, and disability prevention.	73), 80), 81)
Nutrition	Nutrition management	Patients with vascular diseases have malnutrition or hypoalbuminemia, which results in poor postoperative clinical outcomes. Therefore, perioperative aggressive nutritional management is mandatory to improve clinical outcomes.	75), 76), 77), 78), 79), 80), 81)
	Amino acids	Supplementation with amino acids achieves a better body composition and physical activity in elderly patients with frailty or sarcopenia.	82)
Medication	Vitamin D	Vitamin D has the potential not only to prevent falls, but also to increase muscle strength.	84), 85)
	Carnitine	L-carnitine supplementation for frail patients alters their functional status and attenuates fatigue.	89)
Surgery	EVT	EVT by a minimally invasive approach prevents declines in postoperative ADL.	92)
	Hybrid approach	A hybrid approach by combined open surgery and EVT may be useful for high-risk cohorts complicated by vascular disease or with extended vascular disease.	90), 91), 92), 93)
Vascular team approach	Vascular team	Multidisciplinary care for chronic CLI involving vascular, plastic, and podiatric surgeons improved amputation-free survival. A vascular team approach has the potential to enhance quality of care, improve clinical outcomes, and reduce costs.	95), 96), 97)
	Vascular nursing	Vascular nursing may provide comprehensive and optimal care, and achieve better postoperative outcomes.	98)

EVT: endovascular therapy; CR: cardiac rehabilitation; ADL: activity of daily living; CLI: critical limb ischemia

Exercise-based cardiac rehabilitation (CR) may improve clinical outcomes in vascular surgery patients with frailty or sarcopenia. Elderly patients with frailty or sarcopenia generally have few exercise habits in daily life; therefore, clinical symptoms, such as dyspnea on exertion, are more likely. Perioperative CR may contribute to postoperative improvements in physical mobility, functional capacity, fall prevention, disability prevention, or decreased progression and improvements in quality of life following surgical interventions, as reported by Vigorito et al.^73)^ Patients with vascular disease have many preoperative co-morbidities and decreased ADL, particularly patients with CLI; therefore, the early introduction of rehabilitation and mobilization following surgical interventions needs to be promoted and should be made mandatory. Moreover, CR may be a promising strategy for the prevention and treatment of sarcopenia in patients with cardiovascular diseases.^74)^

Nutritional management is also a very important multidimensional intervention for the amelioration of frailty and sarcopenia following vascular surgery. A poor preoperative nutritional status due to chronic undernutrition has been determined to be one of the main pathophysiological mechanisms underlying frailty and sarcopenia, resulting in muscle weakness and functional impairments in daily living.^75,76)^ Moreover, one in four patients who underwent vascular surgery was at risk of malnutrition prior to surgery, which was suggested to increase the risk of developing postoperative complications.^77)^ Wohlauer et al. indicated that since preoperative hypoalbuminemia correlated with higher mortality following endovascular juxtarenal and thoracoabdominal aortic aneurysm repair, it is a useful factor to consider in the assessment of frailty.^78)^ Shiraki et al. demonstrated the importance of the nutrition status on admission in patients with CLI using GNRI.^79)^ They concluded that GNRI on admission was independently associated with mortality and major amputation after EVT in patients with CLI. In addition, amino acid supplementation has been proposed as one of the effective options to achieve a better body composition and physical activity in elderly patients with frailty or sarcopenia.^80)^ Solerte et al. indicated that nutritional supplements with an oral amino acid mixture significantly increased whole-body lean mass in elderly subjects with sarcopenia.^81)^ These findings suggest that improving the preoperative malnutritional status of vascular surgery patients has the potential to reduce the risk of complications and improve the prognosis of these patients. Based on these clinical viewpoints, a comprehensive combinatorial approach with exercise and nutrition for frail patients must be mandatory to improve clinical outcomes in vascular surgical settings.^22,82,83)^

Medication to attenuate frailty and sarcopenia is another important part of the perioperative strategy for these fragile cohorts. Vitamin D has the potential not only to prevent falls,^84)^ but also to increase muscle strength^85)^; therefore, the perioperative intake of vitamin D is a valid option. Testosterone is a typical protein anabolic hormone that is involved in the synthesis of proteins contained in muscle, and it has been shown to promote muscle strength and physical function in frailty cohorts^86)^; however, it does not appear to be a realistic approach due to some of its adverse effects in surgical settings. Statin therapy may promote frailty and sarcopenia because of its adverse effects of muscle injury, namely, statin-mediated muscle dysfunction (so-called statin myopathy), which may underlie sarcopenia.^87)^ Therefore, prolonged medication with statins for patients with sarcopenia needs to be avoided whenever possible. Carnitine is a well-known source of energy production in skeletal muscles, and carnitine deficiency has been associated with aging and contributes to geriatric frailty.^88)^ Badrasawi et al. previously demonstrated that L-carnitine supplementation for frail patients had a favorable effect on the functional status of and fatigue in prefrail older cohorts.^89)^

Minimally invasive surgical approaches may be an alternative option to improve the clinical outcomes of patients with preoperative frailty or sarcopenia. A hybrid approach of open surgery and EVT may be beneficial for high-risk cohorts complicated by vascular disease,^90,91)^ even those with extended vascular disease.^92,93)^ In the current endovascular era, EVT has been increasingly applied to the treatment of various vascular diseases, as it may prevent declines in postoperative ADL.^92)^ EVT may also be aggressively introduced for patients with frailty and sarcopenia.

Some research groups have developed a novel and unique treatment for frailty using stem cell transplantation. This is an innovative approach that is still undergoing clinical trials.^94)^

A vascular team approach will be the ultimate and essential strategy needed to treat frailty and sarcopenia. Chung et al. suggested that multidisciplinary care for chronic CLI involving vascular, plastic, and podiatric surgeons improved amputation-free survival.^95)^ Behrendt et al. promoted multidisciplinary team decision-making, which contributed to the technical success of peripheral vascular interventions and better in-hospital outcomes.^96)^ Kolte et al. recently reported that a vascular team approach has the potential to significantly enhance quality of care, improve clinical outcomes, and reduce costs.^97)^ In addition to a vascular team, vascular nursing has been determined to be an important option that will provide a comprehensive approach to the treatment of patients with various vascular diseases. Ielapi et al. suggested that vascular nursing will provide comprehensive and optimal care, achieve better postoperative outcomes, and facilitate coordinated, standardized, and cost-effective clinical pathways for the management of vascular surgery patients.^98)^

## Limitations

This comprehensive review has some limitations. It does not cover all studies on the clinical implications of frailty and sarcopenia in vascular surgical patients. A meta-analysis is thus needed to provide more precise and significant evidence.

## Conclusion

In vascular surgical settings, the concept of frailty and sarcopenia is now identified to be of great importance in terms of predicting surgical outcomes and promoting appropriate surgical strategies for high-risk cohorts. Thus, to minimize postoperative adverse effects, further clinical research to resolve these current clinical issues and achieve better clinical outcomes following vascular surgical interventions will be deemed mandatory.
